# Global geodiversity components are not equally represented in UNESCO Global Geoparks

**DOI:** 10.1098/rsta.2023.0054

**Published:** 2024-04-01

**Authors:** Emma M. N. Polman, Arie C. Seijmonsbergen, Hannes Versteegh, W. Daniel Kissling

**Affiliations:** Institute for Biodiversity and Ecosystem Dynamics (IBED), University of Amsterdam, PO Box 94240, 1090GE Amsterdam, The Netherlands

**Keywords:** geoconservation, protected areas, global geodiversity, UNESCO Global Geoparks, geodiversity mapping

## Abstract

The aim of UNESCO Global Geoparks (UGGs) is to protect globally significant geoheritage and geodiversity, but quantitative evidence on the global representativeness of geodiversity components (i.e. geology, soils, geomorphology and hydrology) in these geoparks is in short supply. Here, we provide a first assessment by deriving a global map of geodiversity to test whether the presence of geodiversity components in UGGs is representative for the global availability and distribution of geodiversity. Using openly accessible global datasets and a newly developed workflow, we have calculated metrics for each geodiversity component and a global geodiversity index; we then quantified whether UGGs represent global geodiversity and then compared their components to a randomized spatial distribution of geoparks. Our results show that lithological and topographical diversity are more represented in UGGs than outside these sites, while soil type and hydrological diversity are not significantly different. Furthermore, individual soil types and lithological classes are under-represented and unevenly distributed in Asian and European UGGs. This is probably caused by the concentration of geoparks in Asian and European mountains. To better conserve geodiversity, we suggest an initiative to consider the protection and representation of all geodiversity components in their global context.

This article is part of the Theo Murphy meeting issue ‘Geodiversity for science and society’.

## 1. Introduction

Protection of global geodiversity is key to prevent the irreversible loss of valuable information on Earth's scientific and cultural heritage. Natural changes in geodiversity are often slow processes, dating back to the early development of plate tectonics [1]. More recently, geodiversity is rapidly changing through a variety of human activities [2,3]. Such human-induced changes are often related to the (over) exploitation of geosystem services [4,5] provided by geodiversity, for example through the mining of sand, oil, gas and rare earths. In addition, agricultural practices may deplete soil nutrients, urban sprawl leads to covering or leveling of landforms and hydrological interventions such as the erection of dikes, dams and irrigation systems can change river networks. The unsustainable use of geodiversity components means that valuable information about Earth's system processes in the past is often irreversibly lost, owing to the long time scales that are associated with their formation [6–8]. Such changes in the Earth's surface processes are rarely studied or quantified in relation to geodiversity [9], although major progress has recently been made to study relations between geodiversity components [10]. This is supported by the rise of numerous qualitative, quantitative and hybrid assessment methods [11,12]. In addition, the use of essential geodiversity variables for geodiversity assessments has recently been suggested to complement already existing essential climate, ocean and biodiversity variables [13].

From a conservation perspective, geodiversity is included in the concept of World Heritage Sites, UNESCO Global Geoparks (UGGs), some National Parks and other locally protected sites [14]. However, guidelines for including geodiversity are often not formalized or are even absent. Geodiversity and other aspects of environmental heterogeneity create niche spaces and refuge for different organisms, enhancing the coexistence of species and promoting high biodiversity [15,16]. This is supported by strong statistical relationships between geodiversity and biodiversity in different landscapes (e.g. Allahuta *et al.* [17–21]. However, knowledge gaps remain in understanding the spatio–temporal relationship between biodiversity and geodiversity at different scales [22]. To raise awareness for the sustainable use of geodiversity and other of the Earth's resources, ‘International Geodiversity Day’ was proclaimed by UNESCO in 2021 and is now held each year on 6th October [23]. One of the key elements is that geodiversity is fundamental for the implementation of many sustainable development goals, including those associated with biodiversity, human wellbeing and sustainable resource use [24]. For these reasons, the conservation of all components of geodiversity is essential. It is therefore important to evaluate and assess geology, soils, landforms and hydrology across the world and in different landscapes.

Geodiversity studies are increasing in the fields of environmental and conservation science, partly driven by the interest in creating UGGs [25]. A key characteristic of UGGs is that they should host geoheritage and geodiversity of international value [14]; UGGs are defined as ‘single unified geographical regions where sites and landscapes of international geological significance are managed with a holistic concept of protection, education and sustainable development’ [26]. The term ‘geodiversity’ is used in the official description of 41 UGGs (in 2019), often as a synonym for either geoheritage or the presence of unique geological features [27]. All potential sites for UGGs have to apply for membership, based on a wide range of assessment criteria, partly related to geodiversity and its components. One of the assessment criteria of the geopark application procedure is related to the number of rock types, geomorphological features and geological time periods [28]. The occurrence of soil types and hydrological features (e.g. rivers and lakes) and their spatial patterns are not explicitly taken into account, although these are considered important aspects of geodiversity [6] and have been assessed in many studies that quantify geodiversity by using index-based methods [9,29–36]. To date, most geodiversity-related research on UGGs involves qualitative studies and emphasizes their educational and touristic values [37,38]. Quantitative studies of geodiversity in UGGs are rare, especially across broad spatial extents.

**Figure 1.**
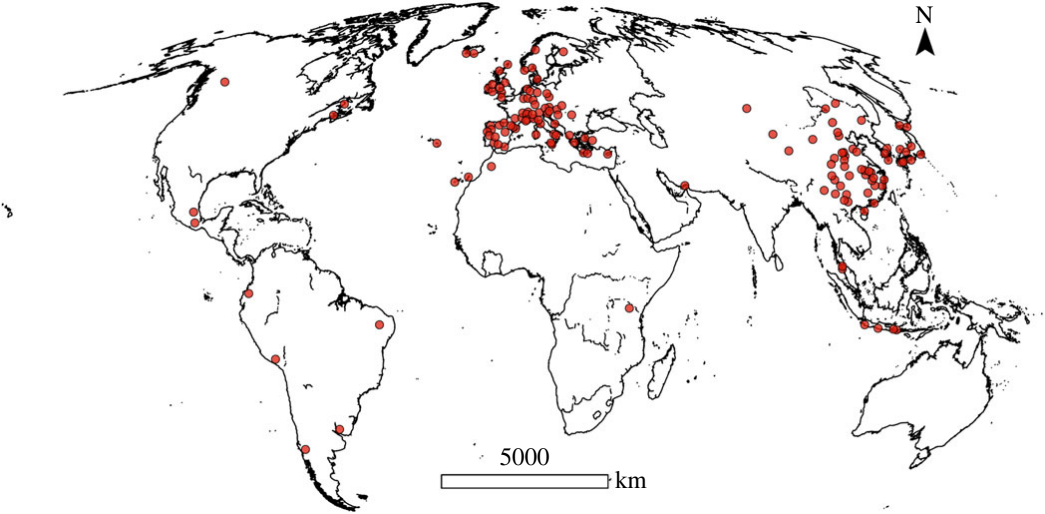
Global distribution of 147 UGGs used for the analysis. Two clusters occur, one in Asia (61) and one in Europe (74). (Online version in colour.)

To date, no systematic research has been conducted to quantify and assess the global representativeness of geodiversity of UGGs. Since the majority of UGGs are located in Europe and China (figure 1), it is unlikely that the entirety of geofeatures on Earth are well represented [10]. Research across 39 UGGs in China suggests that the spatial distribution of the Chinese UGGs is ‘extremely non-uniform’ [39], and significantly affected by government support, tourism and infrastructure, and to a lesser extent by landform and the distribution of geosites. Similarly, for the European continent UGGs have been reported to be unevenly distributed [36]. For a global assessment, considering the wide variety of landscapes, a standardized method to quantify geodiversity across large areas is necessary [36,40,41]. Over regional and national extents, index-based approaches have been developed, in which thematic maps (e.g. soils, geomorphology and hydrology) are used as inputs to count unique geofeatures per cell, which results in the documentation of the variety of geodiversity [9,42–44]. However, such maps do not yet exist on the global scale.

Here, we describe the development of a global map of geodiversity and test to what extent global geodiversity and its components are well represented in UGGs. We focus on 147 regions that have been awarded the status of Global Geopark by UNESCO between 2001 and 2019. We have developed a global geodiversity index, based on openly accessible geospatial layers of geodiversity components. Specifically, we (1) calculated global layers with scores of lithological, pedological, topographical, hydrological diversity as well as total geodiversity and (2) compared the diversity scores of these layers in UGGs with randomly selected areas of comparable size outside the UGGs. Our analysis identifies those aspects of global geodiversity that are under-represented in current geoparks and provides a workflow that can assist in selecting other areas that are potentially worth protecting in a global context.

## 2. Material and methods

**Figure 2.**
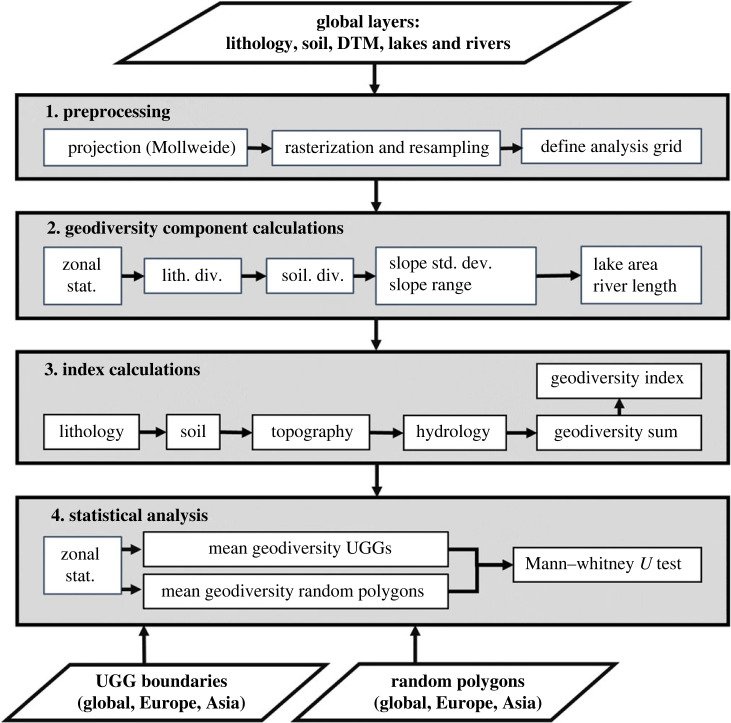
Workflow for comparing global, Asian and European geodiversity (component) scores in and outside UGGs. The workflow has four routines: 1. Preprocessing of global layers, 2. Geodiversity metric and component calculations, 3. Index calculations and 4. Statistical analysis. Input layers are indicated in parallelograms.

Our developed workflow (figure 2) includes four main routines to assess the representativeness of global geodiversity in UGGs, namely (1) preprocessing of the input layers, (2) geodiversity metric and component calculations, (3) index calculations for geodiversity components and global geodiversity and (4) statistical analysis to test whether UGGs represent areas with high scores of geodiversity metrics and total geodiversity. The workflow required input datasets, i.e. global input layers to quantify each geodiversity component (shown as parallelogram in the top part of figure 2) and polygons describing UGG distributions (parallelograms below the workflow steps in figure 2). In the workflow, we used the model builder in ArGIS Pro [45] for preprocessing, geodiversity metric and component calculations and index calculations. The getJenksBreaks function in R [46] was used to classify the final geodiversity index into five classes (very low, low, moderate, high, very high). We used the ArcPy module in Python 3.6.8 [47] to generate random polygons, and to calculate the mean geodiversity metric scores in these random polygons. The pandas [48], SciPy [49] and Statsmodels packages [50] were used for the statistical analyses of the resulting geodiversity component scores, and the Matplotlib [51] and seaborn packages [52] were used for data visualization.

**Table 1.** Details of the input data used to create global geodiversity metrics and components for a 10 × 10 km grid.

			scale/			
			(cell) size		geodiversity	geodiversity
dataset	description/source	reference	resolution	format	metric	component
GLiM	Global Lithological Map Database v1.0 (gridded to 0.5° spatial resolution) (pangaea.de)	Hartmann & Moosdorf [53]	average 1 : 3 750 000	vector- Polygon	variety of 15 first-level lithological types	geology, i.e. lithological diversity
SoilGRIDS	global soil types—SoilGrids250m 2.0	Hengl *et al*. [54]	250 m	raster	variety of 30 first-level soil types	soils, i.e. soil type diversity
MERIT DEM	global elevation data—MERIT DEM: multi-error-removed improved-terrain DEM (u-tokyo.ac.jp)	Yamazaki *et al*. [55]	3 arc-seconds	raster	1. Standard deviation of slope (degrees) 2. Slope range (degrees)	geomorphology, approximated with topographical diversity
GLoRiC	global river data—GloRiC (hydrosheds.org)	Dallaire *et al*. [56]	—	vector- line	river length (km)	hydrology, hydrological diversity
HydroLAKES	global lake boundaries—https://www.hydrosheds.org/products/hydrolakes	Messager *et al*. [57]	lakes and dams >10 ha	vector- polygon	lake area (km^2^)	

### (a) Global layers

We preprocessed five published, downloadable and harmonized open-access global layers reflecting lithology, soil, terrain, rivers and lakes (table 1). These datasets were used to represent the four major geodiversity components [13,58], namely geology, soils, geomorphology and hydrology (table 1).

For the geology component, we used the Global Lithological Map (GLiM) created by Hartman & Moosdorf [53] (table 1), which was constructed from ninety-two national and/or state agency geological maps in polygon format. We used the first legend level to calculate the geology component, i.e. the number of lithological types. In this level, formation names or individual rock types (e.g. sandstone, basalt) have been combined to a broader characterization of lithological units, which reflect the variety of major rock (sedimentary, metamorphic, igneous) domains. Although details of specific mineralogical composition or fossil content are not captured in this first legend level, the second and third legend levels could not be used because they lack the necessary global coverage. From the available 16 first-level legend entries, the following 15 lithological types were used (the category ‘No Data’ was discarded): ‘unconsolidated sediments' (US), ‘siliciclastic sedimentary rocks’ (SS), ‘mixed sedimentary rocks' (SM), ‘carbonate sedimentary rocks’ (SC), ‘pyroclastic rocks’ (PY), ‘evaporites’ (EV), ‘metamorphic rocks’ (MT), ‘acid plutonic rocks’ (PA), ‘intermediate plutonic rocks' (PI), ‘basic plutonic rocks’ (PB), ‘acid volcanic rocks’ (VA), ‘intermediate volcanic rocks' (VI), ‘basic volcanic rocks’ (VB), ‘ice and glaciers’ (IG) and ‘water bodies’ (WB). It should be noted that this first level provides a generalized characterization of lithological units, in which the diversity of metamorphic rocks is clustered in one category whereas plutonic rocks have been separated into different categories.

For the soil component, we used information from the updated SoilGrids250 (table 1) repository [54]. This repository contains a grid-based map of 18 unique soil types, classified according to the World Reference Base (WRB) classification scheme [59]. In addition, numerous soil-chemical (e.g. pH, CEC) and soil-physical (e.g. textural variation, soil organic carbon) layers at various depths (between 0 and 200 cm) are available, which form a wealth of detailed geodiversity information. The global data are the result of using more than 150 000 harmonized soil profiles together with remote sensing-based covariates to fit an ensemble of machine learning methods for predicting soil information in raster cells with 250 m resolution with a global extent [54]. We here used the 30 first-level soil types because their classification reflects a variety of soil-chemical and soil-physical processes, and soil types are comparable with the first-level lithological types.

The geomorphology component was approximated with topographical information (table 1) because a globally harmonized geomorphological map is currently not available. We therefore used the Multiple-Error-Removed Improved-Terrain (MERIT) Digital Elevation Model (DEM) [55] as the input (table 1). This elevation model introduces the possibility to consistently calculate terrain metrics that reflect topographical variation (e.g. variability in slopes and elevation). Even though it misses genetic information about landforms, which is thought to be essential in geomorphological maps [60,61], the use of DEMs as a surrogate for geomorphology has proven successful at landscape-scale evaluations with geodiversity indices [9,62]. The MERIT DEM has a resolution of three arc-seconds, which is equal to approximately 90 m at the equator, and is based on a combination of multiple DEMs [55]. The MERIT DEM was used to calculate the standard deviation of slope and the range of slope to represent topographical variation across the globe. We expect these two parameters to reflect topographic surface roughness and topographic heterogeneity in a landscape.

For the hydrology component, we used two datasets (table 1), namely the global river classification (GLoRiC) dataset [56] and the lakes dataset of the world [57]. The GLoRiC dataset is based on a conceptual framework in which 127 global river types are classified, and is accessible as vector data. For our global geodiversity assessment, we used the river vectors to calculate river length (a hydrometric parameter) as the input for our analysis. Lakes were extracted from the global HydroLAKES dataset, in which 1.43 million individual natural lakes and human-made reservoirs larger than 10 ha are mapped [57]. In addition, the dataset contains information on area, lake area density, shoreline length, water volume and average depth. For our global geodiversity assessment, it was sufficient to use the lakes to calculate lake area (a hydrometricparameter).

### (b) Preprocessing

The various input datasets came with different spatial resolutions and formats (table 1); they were originally prepared for different purposes and using different techniques. For use in a comparative index-based study, it is essential to ensure that the input layers have comparable map scales, that legend categories are harmonized across countries and that redundant map information is removed. This enables an objective calculation and statistical comparison of the global distribution of geodiversity and geodiversity components inside and outside of UGGs. In the first workflow routine (figure 2), we therefore projected the input layers on to an equal area Mollweide projection, rasterized and resampled them to a resolution of 125 m grid cells, and used a 10 × 10 km grid for subsequent analysis. The choice of the analysis grid was guided by a simple formula (scale number × 25) to objectively select an analysis grid when using maps of different scales and cell sizes [63]. In our case, we used the coarsest scale number (3 750 000, table 1) and rounded to 10 km. All results are presented at the 10 × 10 km resolution.

### (c) Geodiversity metric and component calculations

In the second workflow routine (figure 2), zonal statistics were calculated for each preprocessed input dataset using the 10 × 10 km analysis grid. The geodiversity components included the lithological diversity, the soil type diversity, the topographical diversity and the hydrological diversity (figure 2). The topographical diversity included metrics of the standard deviation and range of the slope, to optimally reflect the morphometrical variation of landforms across altitudinal gradients. The hydrological diversity combines river length and lake area metrics in the 10 × 10 km grids, to effectively reflect the presence and variations in surface waters. The soil type diversity and lithological diversity were calculated within a 10 × 10 km grid cell as the number of unique soil types and lithology types, respectively.

### (d) Index calculations

In the third workflow routine (figure 2), all geodiversity component layers were converted into an index, i.e. reclassified into five classes (1. very low, 2. low, 3. moderate, 4. high, 5. very high) using a Jenks natural-breaks classification. The total geodiversity layer was then calculated as the sum of all individual diversity scores (total geodiversity = lithological diversity + soil type diversity + topographical diversity + hydrological diversity). The geodiversity index map is thus a categorization of total geodiversity using a Jenks natural-breaks division into the five geodiversity index classes described above, to support optimal visualization.

### (e) Statistical analysis

A geodatabase was constructed that contained the 147 UGGs listed on the UGG Network website (2019) and the UNESCO website (2021). Because UNESCO is not allowed to share its database, the outlines of the park areas from the UGGs websites were manually digitized from available maps or via email requests to the park authorities. For parks that were not covered (*n* = 40, 27%), the coordinates and areas of the UGGs provided by UNESCO [64] were used to create circular buffers, equal to the particular park area. In two cases part of the circular polygons that overlapped with sea or ocean surfaces were removed. Electronic supplementary material, table S1 lists an overview of all UGGs used in the analysis.

To test whether the 147 UGGs (figure 2) represented areas with higher geodiversity (metric) scores, we first used zonal statistics to calculate the mean of the six geodiversity metrics (i.e. variety of lithological types, variety of soil types, standard deviation of slope, slope range, river length and lake areas), and the mean of the total geodiversity in each UGG (figure 2). Thereafter, a random distribution of UGGs was simulated by randomly placing 147 circular polygons with a similar surface area distribution as the UGGs across the Earth's surface. For this random sample, the mean of the six geodiversity metrics and the mean total geodiversity were calculated. These represent the mean geodiversity one would expect if UGG locations were chosen randomly, and not to represent high geodiversity or geoheritage values. Because of the non-normality of the data, a Mann–Whitney *U* test was used to test if the mean geodiversity metrics and total geodiversity were higher in UGGs than in the random sample. To ensure the robustness of the analysis, this procedure was repeated 100 times. Performing a test multiple times increases the family-wise error rate, the *p*-values were therefore corrected using the Holm–Sidak method. This method corrects for multiple testing while conserving statistical power [65]. The World Countries dataset from ESRI [66] was used to repeat this entire analysis on the Asian and European extent, using 61 and 74 UGGs, respectively (figure 2).

## 3. Results

### (a) Global layers

**Figure 3.**
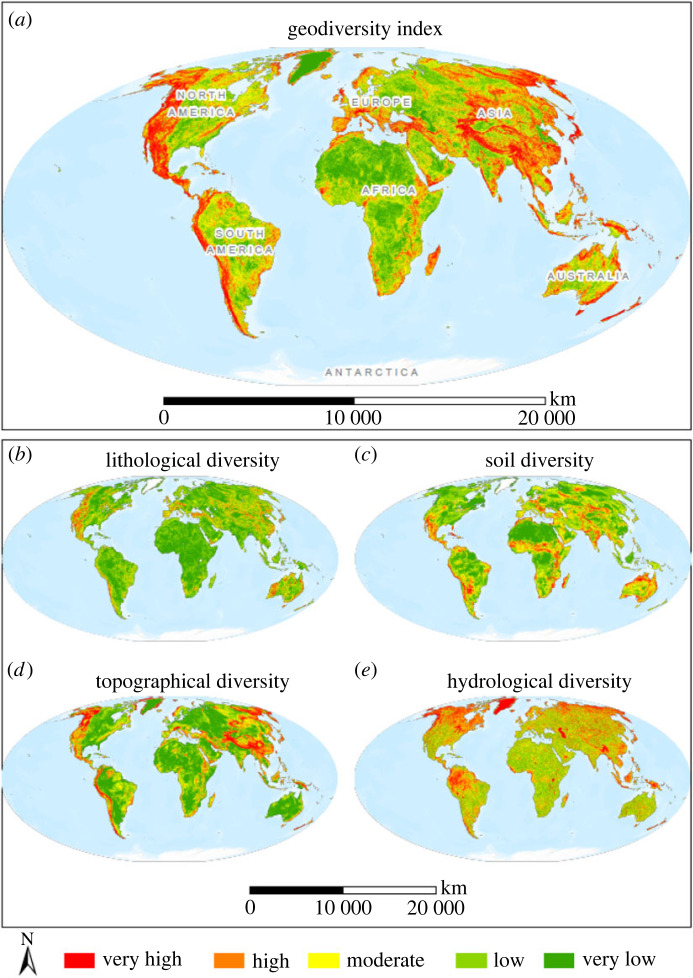
Global distribution of geodiversity. (*a*) Global geodiversity index, (*b*) lithology diversity index, (*c*) soil diversity index, (*d*) topographical diversity index and (*e*) hydrological diversity index. For visualization and comparison purposes, all data are presented in five classes (1. very low, 2. low, 3. moderate, 4. high, 5. very high) using a Jenks natural-breaks classification.All maps are available for download via figshare: https://doi.org/10.21942/uva.23496923. (Online version in colour.)

Our global geodiversity index map shows that very high to high geodiversity is found in (formerly) active tectonic zones such as mountain ranges, island arcs and rift zones, for instance in the Andes, the Rocky Mountains, the European Alps, the Himalayas, the Indonesian archipelago, the African rift zone and the Mediterranean basin (figure 3*a*). Relatively low to moderate geodiversity occurs in the stable cratons of South America, Africa, India and Australia, in formerly glaciated areas in the Northern Hemisphere, and in areas with intermediate hilly relief on all continents. Very low geodiversity is predominantly found in the downstream parts of the world's major river drainage basins (e.g. the Amazon, Mississippi, Yamuna-Brahmaputra and Nile), in large deserts (e.g. Sahara, mid-Australia) and on ice caps (e.g. Greenland). All datasets, i.e. the geodiversity metric layers, the geodiversity component layers and the total geodiversity layer, are openly available for download at doi:10.21942/uva.23496923.

The distribution of lithological diversity reflects the variety of rock formations, which is high in areas with relatively complex structural deformation (e.g. in mountains and plate collision zones), and low in areas that are relatively large and tectonically stable, and/or in descending basins that receive large quantities of sediments (e.g. large river deltas, or floodplains) (figure 3*c*). The global distribution of soil diversity shows (figure 3*b*) high index values in mountainous areas (azonal soil formation), and low index values in spacious, relatively low elevated areas with a continental climate, e.g. in mid-western Asia and the USA (zonal soil formation). Topographic diversity is high in areas with high relief energy, characterized by a large elevation range and steep slope angles; in principle in all young (e.g. Alpine, Hercynian and Caledonian) and old (e.g. Laurentian and Algoman) mountains, in dissected low- and high-elevated plateaus, and on large volcanoes (figure 3*d*). Hydrological diversity is particularly high in formerly glaciated areas of the northern hemisphere (generally above 50 degrees North) with deranged hydrological patterns (figure 3*e*), for instance in Canada, Scandinavia and northern Russia, as well as in tropical areas that receive high amounts of precipitation, such as the Amazon basin and the large islands between SE Asia and Australia (e.g. Borneo and Java).

### (b) Representativeness

**Figure 4.**
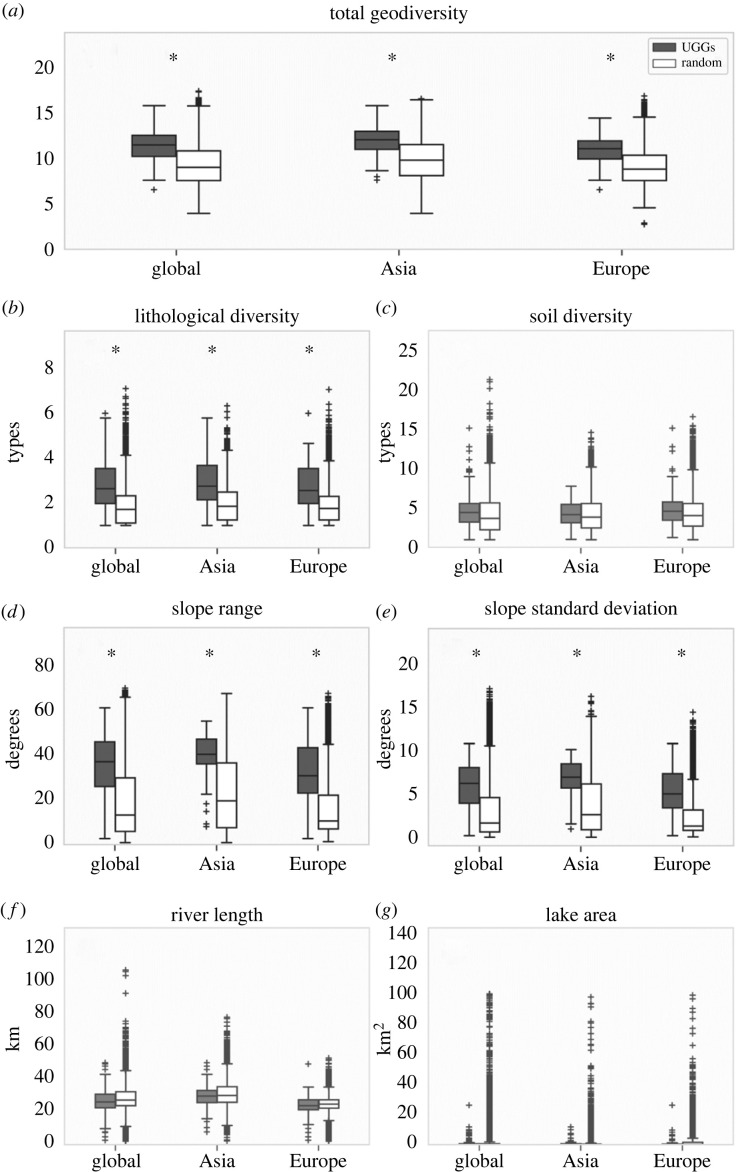
Boxplots displaying the distribution of total geodiversity and geodiversity metrics within UGGs and outside geoparks (in randomly selected polygons), separately shown for a global, Asian and European extent.(*a*) Total geodiversity and (*b–g*) individual geodiversity metrics (see details in table 1). ^*^ Indicates that the metric is significantly higher (corrected *p* < 0.05) in UGGs then in the random samples for all 100 comparisons. An overview of the percentage of corrected *p* < 0.05 for each metric is available in electronic supplementary material, table S3. For visualization purposes, the data of all 100 random sampling repetitions are shown together in one boxplot.

On a global scale, total geodiversity is significantly higher in UGGs compared to random placements of geoparks across the world. This result is also found when focusing the analysis on UGGs in Asia and Europe (figure 4*a*). Overall, the mean total geodiversity of random samples ranges from 9.1 to 9.9, while the mean total geodiversity in UGGs ranges from 11.1 to 12.0, on all extents. Electronic supplementary material table S2 lists an overview of the mean total geodiversity and mean geodiversity metrics in UGGs and random samples on global, Asian and European extents.

The lithological diversity metric (figure 4*b*) is significantly higher in UGGs compared to random placements of geoparks on the global, Asian and European extents. A comparable result is found for the standard deviation of slope and the slope range metrics (figure 4*d,e*). By contrast, soil diversity and hydrological diversity (including both lake area and river length) do not differ significantly (figure 4*c,f,g*). The mean standard deviation of slope angle scores ranges between 2.4 and 3.7 degrees in random samples, and between 5.4 and 6.7 degrees in UGGs. A similar difference is apparent for the mean slope ranges (15.3–21.8 degrees in random samples versus 31.8–38.3 degrees in UGGs). The mean scores of the lithological diversity range from 1.9 to 2 types/100 km^2^ in random samples and from 2.7 to 2.9 types/100 km^2^ in UGGs. The mean river length is approximately 2 km shorter in UGGs compared to scores computed for the global extent, and approximately 1.5 km shorter for the scores computed for the Asian and European extents. Lake areas range, on average, between 1.01 × 10^6^–1.20 × 10^6^ in UGGs and between 1.35 × 10^6^–2.22 × 10^6^ in random samples on global, Asian and European extents.

## 4. Discussion

A global map of geodiversity and its components has been developed, and evaluated on whether geodiversity is representative in UGGs compared to a random placement of geoparks. Our results show that lithological and topographical diversity are significantly higher within UGGs, but that soil and hydrological diversity are not significantly different. This suggests that not all soil and lithology types are equally represented in UGGs, which is probably because of UGGs being concentrated in the mountainous regions of Asia and Europe.

### (a) Representativeness of geodiversity in UGGs

The present spatial distribution of global geodiversity and its components reflect the long natural history of our planet. It is the result of variation and intensity in endogenic and exogenic processes, and influenced by variation in climate on global, regional and local scales. Our global analysis showed that areas with UGGs (less than 1% of the global terrestrial surface) represent areas with higher lithological and topographical diversity than randomly selected areas outside UGGs. This can probably be explained by the majority of UGGs (59%) being located in mountains, while only 12% of the global terrestrial surface is considered mountainous [67]. On a global scale, the areas with the highest total geodiversity values also occur in mountains (figure 3). This can be largely attributed to the higher variation in lithological diversity and topographical diversity and supports the idea that mountains are hotspots of geodiversity [67,68]. Mountains also support high biodiversity [18], suggesting the two are tightly linked (‘mountain-geobiodiversity hypothesis'; [21]). Interestingly, Testolin *et al*. [69] mention that there are biodiversity ‘hotspots’ and ‘coldspots’ among different alpine ecosystems which may require a more detailed exploration of the relationship between species diversity, topographical diversity and soil diversity. The high and unique biodiversity in mountains [18,21,70] could also be used to promote the fact that these areas are of great interest in the UGG selection procedure. However, other factors such as government support, tourism and infrastructure also play an important role in selecting UGGs [39].

The lithological diversity inside UGGs is higher in Europe, Asia and globally compared to randomly selected areas within those extents. The location of UGGs in mountains contributes to this pattern because mountains reflect complex geological developments including compressive crustal deformations which lead to a large lithological variation over relatively short distances. Given this high lithological variation, it is plausible that during the application procedure for becoming a UGG specific emphasis is put on selecting rock types in which distinct geological features are observed. The high aesthetic value of spectacular and well-exposed rock formations and tectonic structures may make mountainous areas especially popular for UGG establishment [14]. Our lithological diversity metric captures the variety of rock types, but does not take into account which rock types are actually present in UGGs. An additional measure of the representation of lithological diversity could be the area covered by each rock type within UGGs compared to its total area within a given region (e.g. in Asia and Europe). Such an analysis shows that especially igneous rocks (e.g. intermediate plutonic rocks, 11% covered in UGGs) and volcanic rocks (e.g. Pyroclastics, 18% covered in UGGs) are relatively well represented in European UGGs (electronic supplementary material, table S4). This is in agreement with earlier, qualitative studies that concluded that volcanic geoheritage is well represented in geoparks (Liu *et al.* [14,71]). The percentages for sedimentary rocks in European UGGs (e.g. carbonate sedimentary rocks, 2.3%) and metamorphic rocks (1.9%) are substantially lower (electronic supplementary material, table S4). This can be partly explained by the relatively large surface area covered by sedimentary rocks in Europe (electronic supplementary material, table S4). For Asia, all rock types have a low percentage of coverage in UGGs (≤0.5%) because Asia covers a huge area compared to the area of the UGGs (electronic supplementary material, table S4).

We found that soil diversity in UGGs is not significantly different from randomly placed areas outside UGGs. This may be explained by the large share of UGGs in mountainous areas. However, soil formation is not only driven by differences in relief, but also by differences in climate (temperature and rainfall), organisms, parent material and the time available for soil formation [72]. The dynamics and interrelationships between soil diversity and soil forming factors are therefore complex [25]. Previous studies have reported that lower soil diversity is associated with extreme temperatures and precipitation [73], and increasing elevation [74]. In mountainous and hilly areas of the humid tropics and subtropics, soil diversity is highly influenced by formation time, and the parent material has more influence on diversity at low elevations [75]. The location of most UGGs in mountainous areas also suggests that not all soil types are equally represented in UGGs. Considering that mountain soils are in general azonal and relatively young [76], we expected that zonal soil types, and soil types associated with long formation times are less represented in currently designated UGGs. A calculation of the total surface areas of soil types and their representation in Asian and European UGGs shows that andosols are especially well represented in European UGGs (greater than 35% area covered; electronic supplementary material, table S5). This also reflects the high presence of volcanic rock types (electronic supplementary material, table S5). However, not all soil types that occur in Asia and Europe are represented in Asian and European UGGs. In Asian UGGs, soils such as durisols, solonetz, umbrisols, albeluvisols and stagnosols are not represented at all (electronic supplementary material, table S5). Similarly, ferralsols, gypsisols, planosols, nitisols and solonetz do not occur in European UGGs (electronic supplementary material, table S5). Many of these soils are zonal soils, restricted to specific climatic zones, or soils that require long formation times. To date, soil types or landforms related to soil formation are not explicitly included in the UGG application criteria. Our analyses imply that areas of high soil diversity or soils with low areal cover in mountains are currently underrepresented in the UGG site network. This emphasizes the need of establishing UGGs in all climatic zones, in order to better capture the global diversity and distribution of soils.

The topographical diversity, here approximated with the standard deviation and range of terrain slopes (table 2), is significantly higher in UGGs than in areas outside UGGs. This is a direct consequence of nearly 60% of the UGGs being located in mountains. Although DEM-derived metrics are used in other studies as alternatives for geomorphology, it hampers the evaluation of landforms in UGGs relative to other areas. The variety of landforms occurring in high elevated mountains (e.g. glacial, and mass movement-related landforms) can be high, but landform variety in low-angle areas can also be high (e.g. fluvial, aeolian and marine landforms, [77]). This is not seen when using DEM-derived metrics alone.

The hydrological diversity is slightly lower in UGGs compared to areas outside. Ruban [78] analysed the official descriptions of 144 UGGs in order to understand the involvement of water by geopark creators/managers, and found that only 55% mention water, with 47% of those showing photographs of water objects, mainly to stress the aesthetic value. Our analyses suggests that the low hydrological diversity in UGGs can be explained by the overrepresentation of UGGs in mountains, as the hydrology in mountainous areas often consists of small and fragmented streams. In river-dominated lowland areas and in formerly glaciated continental areas the drainage patterns contribute to large lake areas and long rivers, which are well captured in global hydrological datasets [56,57]. By contrast, mountainous areas only develop small hydrological networks, which are not well represented in global river datasets, although the number of streams in mountains can be higher than in lowland situations. A similar situation exists in relation to lakes, especially in high mountain environments. For example, cirque lakes, paternoster lakes, former ice-dammed lakes and small artificial lakes smaller than 10 ha are not included in the global lakes dataset.

Our study focuses exclusively on UGGs, but other (local, national) protected areas may already include areas with higher soil and hydrological diversity and larger areas of the underrepresented soil and/or lithology types. It would be interesting to expand the testing of our workflow to evaluate the representativeness of geodiversity on a global scale by including new UGGs, other protected areas or nature reserves of national and/or regional importance such as the natural sites from the World Heritage list. Advancements in harmonizing criteria to objectively include relevant geodiversity components would also benefit future assessments.

### (b) Methodological aspects

Our methodology uses harmonized and openly accessible datasets with global coverage to create geodiversity metric layers and a total geodiversity layer with a global extent (figure 2). While this is, to our knowledge, the first global study of its kind, a number of methodological aspects can be highlighted to guide future studies and improvements.

For lithological diversity, the GliM [53] data does not explicitly include layers of mineralogical and fossil content to construct a geodiversity assessment following the definition of geodiversity by Gray [58]. Such layers have been included in a number of regional or national studies in index-based geodiversity assessments (e.g. [30,79]). However, fossils mostly occur in sedimentary rocks such as limestone, and mineralogical associations differ greatly between sedimentary and magmatic rock types. Therefore, we expect that sufficient variety is embedded in the highest legend category. Lower levels of classifications could include, if available, refined subdivisions of lithological diversity to better reflect regional and local variations. For example, different metamorphic rocks are now summarized in one legend category, which could influence their representation in the UGGs compared to the three plutonic rock categories which are separately represented. Regarding the quality of the underlying expert-based lithology maps, it is important to mention that these maps differ in their original scale. Some were made in difficult-to-assess areas, which can influence their quality and consistency, despite the development of harmonized legends.

For soil diversity, the soil type classes are based on detailed variations in texture and other soil physical and chemical characteristics, which have been identified by soil experts from hand drillings. As such, they reflect the soil types embedded in the highest legend category of the SoilGrids database [54]. Similar to lithology, detailed refinements are possible at lower level subdivisions, and applicable at regional or local extents. We expect future improvements of digital soil maps if harmonization with national, regional and local soil drilling programmes progress further and if new, more sophisticated algorithms are developed. Global geodiversity evaluations can then be refined accordingly.

We approximated geomorphology to topographical diversity. Although a legend for global geomorphological mapping has existed for more than 50 years [80], a global map is still not available because the scientific community continues to use national mapping systems [81]. The global DEM-derived morphometric alternatives in our analyses offer a realistic proxy for geomorphology. Future analysis of global geodiversity could include landform elements (e.g. peaks, ridges, footslopes, hollows, valleys, shoulders, pits, slopes, flats and spurs), represented by geomorphons [82,83] to add a larger variety of landform elements. When using such datasets, it is important to include the genesis of landforms, as this is a crucial element of geomorphology. Harmonization of national and regional geomorphological maps, perhaps in combination with geomorphons, is necessary to fully include geomorphology in a global geodiversity map. Geomorphology is already included in a global geomorphological map of the ocean floor [84], which has been used in an index-based inventory of geodiversity of the Pacific Ocean [85].

We opted to, in equal measure, include lake area and river length as metrics in the calculation of hydrological diversity, and considered a total coverage by water to represent a very high class. Part coverage of a cell by a lake area, combined with variable river lengths, could result in very low to very high hydrological diversity. Other choices could be made to represent hydrological diversity. For instance, future refinements could consider to include a topographic wetness index to calculate hydrological diversity (e.g. applied by Najwer *et al*. [86] in national parks in Poland) or, alternatively, to include river drainage order classifications [87,88]. Higher resolution DEMs, e.g. based on high-resolution LiDAR campaigns such as those from the GEDI mission [89], can also advance the quantification of geodiversity metrics at fine resolutions over a large spatial extent, for example by allowing better detection of first-order streams in mountains which are underrepresented in the currently available HydroSheds dataset [90]. Additional information on artificial dams, drainage and irrigation channels [77,91,92] or groundwater, springs, wetlands and other ‘water-related’ geofeatures could be relevant to include for capturing hydrological diversity globally and within UGGs [78].

## 5. Conclusion

Our study demonstrates that the representation of global geodiversity components in UGGs can be evaluated by using openly accessible datasets of (1) harmonized thematic layers of soil types and lithology types, (2) hydrological data of river lengths and lake areas and (3) topographical metrics calculated from DEMs. The results show that the variety of lithology types and areas with relatively steep slopes and elevation ranges are significantly more represented in UGGs than in randomly selected areas outside UGGs. This is probably caused by the uneven spatial distribution of UGGs which are concentrated in Asia and the EU, specifically in mountains. On the other hand, hydrological and soil diversity do not significantly differ in and outside UGGs. Volcanic rock types and soils developed on volcanic parent materials are most represented in UGGs. In relation to volcanic parent materials, andosols are most abundant, while soils associated with (semi-) arid climates are currently underrepresented in UGGs. To better conserve geodiversity at a global scale, we recommend to consider including all components of global geodiversity in a representative network of geoparks across the world. We also urge future assessments of geodiversity, not only at global but also local, national and regional scales.

## Data Availability

The digital data are available via figshare: Polman, EMNP; Seijmonsbergen, ACS; Versteegh, Hannes; Kissling, WDK [93]. Dataset - Is global geodiversity represented in UNESCO Global Geoparks? University of Amsterdam. Dataset. https://doi.org/10.21942/uva.23496923 [94]. Supplementary material is available online [95].
